# Provider perspectives on the acceptability and tolerability of dolutegravir-based anti-retroviral therapy after national roll-out in Uganda: a qualitative study

**DOI:** 10.1186/s12879-021-06933-8

**Published:** 2021-12-07

**Authors:** Henry Zakumumpa, Freddy Eric Kitutu, Helen Byomire Ndagije, Nakitto-Kesi Diana, Jacquellyn Nambi Ssanyu, Ronald Kiguba

**Affiliations:** 1 School of Public Health, Makerere University College of Health Sciences, Kampala, Uganda; 2 Sustainable Pharmaceutical Systems (SPS) Unit, Pharmacy Department, School of Health Sciences, Makerere University, Kampala, Uganda; 3 National Drug Authority, Directorate of Product Safety, Kampala, Uganda; 4 Department of Pharmacology and Therapeutics, Makerere University College of Health Sciences, Kampala, Uganda

## Abstract

**Background:**

The World Health Organization recommends dolutegravir (DTG) as the for first-line and second-line antiretroviral therapy (ART) worldwide. However, little is known about the acceptability and tolerability of DTG-based ART at routine points-of-care in Uganda. We set out to explore the perceptions of clinicians in ART clinics regarding the acceptability and tolerability of DTG-based ART since national roll-out in March 2018 in Uganda.

**Methods:**

We adopted a qualitative exploratory design involving 49 participants. Between September 2020 and February 2021, we conducted 22 in-depth interviews with clinicians in the ART clinics of 12 purposively selected health facilities across Uganda. The selection of study sites ensured diversity in facility ownership-type (public/private), level of service delivery (tertiary/secondary/primary) and the four major geographic sub-regions of Uganda. We conducted three focus group discussions with 27 clinicians in the participating facilities. Data were analyzed by thematic approach.

**Results:**

Clinicians in ART clinics acknowledged that DTG-based ART is well tolerated by the majority of their patients who appreciate the reduced pill burden, perceived less side effects and superior viral load suppression. However, they reported that a number of their patients experience adverse drug reactions (ADRs) after being transitioned to DTG. Hyperglycemia is, by far, the most commonly reported suspected ADR associated with DTG-based regimens and was cited in all but two participating facilities. Insomnia, weight gain and reduced libido are among the other frequently cited suspected ADRs. In addition, clinicians in ART clinics perceived some of the suspected ADRs as resulting from drug interactions between dolutegravir and isoniazid. Weak diagnostic capacities and shortage of associated commodities (e.g. glucometers and test kits) were reported as impediments to understanding the full extent of ADRs associated DTG-based ART.

**Conclusion:**

While DTG-based regimens were perceived by clinicians in ART clinics to be well tolerated by the majority of their patients, they also reported that a number of patients experience suspected ADRs key among which were hyperglycemia, insomnia and reduced libido. Based on the perspectives of clinicians, we recommend that future studies examine the prevalence of dolutegravir-induced hyperglycemia in patients in Uganda.

**Supplementary Information:**

The online version contains supplementary material available at 10.1186/s12879-021-06933-8.

## Background

Universal access to anti-retroviral therapy (ART) is enshrined in the Sustainable Development Goals (SDG 3.3) and in UNAIDS’s 95-95-95 targets for ending AIDS as a public health threat by 2030 [1]. Globally, there are 37.9 million people living with HIV [1]. Out of these, 24.5 million are currently accessing ART [1].

Following evidence from clinical trials that demonstrated that DTG-based ART had superior patient outcomes compared to alternative combinations (such as those containing efavirenz) [2, 3], the World Health Organization (WHO) revised and published guidelines recommending dolutegravir (DTG)-based ART as the first-line regimen in HIV treatment [2, 3].

In addition, there are studies suggesting that DTG may result in cost savings in procurements of antiretrovirals especially in countries in sub-Saharan Africa which are heavily dependent on international assistance for their national HIV responses [4, 5].

In Uganda, DTG-based ART was rolled-out nationally as the recommended first-line regimen starting in March 2018 following this WHO guidance [2, 3]. President’s Emergency Plan for AIDS Relief (PEPFAR) the predominant HIV donor in Uganda [6] included DTG roll-out in its annual program targets for the national HIV response in Uganda [7]. Following PEPFAR programmatic guidance, procurement of ART commodities in Uganda was aligned accordingly. PEPFAR and The Global Fund are the leading sources of finance in the procurement of antiretrovirals in Uganda [6].

In 2019, an aggressive 100-day roll-out of anti-tuberculosis medication known as isoniazid preventive therapy (IPT) was implemented across Uganda [8]. Tuberculosis is the leading cause of death of people living with HIV (PLHIV) [9]. Tuberculosis preventive therapy has been shown to reduce mortality in PLHIV [9].

Against the backdrop of the introduction of DTG-based ART as the new recommended first-line regimen [2] and the aggressive roll-out of IPT across Uganda [8], patient and medication safety concerns may have been overlooked [10]. The ambitious roll-out of these HIV care and treatment strategies were not accompanied by commensurate investments in robust pharmacovigilance systems. There is a dearth of empirical data exploring medication-related harms despite growing patient complaints of the safety profile of these newer HIV medications [11]. This development is in spite of the fact that anti-retroviral medicines contribute the highest number of case reports of adverse drug reactions (ADRs) of any medication in Uganda [10]. DTG-based ART is associated with an increase in the Body Mass Index of users (or becoming obese/ overweight) [12] and has come under the spot light following safety signals of neural tube defects in infants born to women exposed to DTG in the first 3 months of conception [13]. Studies conducted in Uganda and South Africa have explored the perceptions of women around the use of DTG-based ART during pregnancy [14–16]. The acceptability of new medicines or interventions is influenced by a diverse range of factors that include socio-cultural beliefs, gender dynamics, socio-economic status and health system constraints [17–19].

Despite the critical importance of preventing medication harm, there is little research documenting the safety profile of newer HIV medications in Uganda following national roll-out of DTG-based ART in 2018. Most of the evidence base on the safety profile of DTG has been generated from clinical trials [2–4]. The safety profile of DTG-based ART in ‘real-life’, routine points-of-care in countries with a high HIV burden such as Uganda is not adequately understood [20–22].

We set out to explore the perceptions of ART clinic managers in Uganda regarding the acceptability and tolerability of DTG-based ART following national roll-out in March 2018.

## Methods

### Study design

A cross-sectional qualitative exploratory research design was adopted [23]. We sought to understand the perspectives of clinicians in the ART clinic regarding the safety and tolerability of DTG-based ART based on their occupational experiences of attending to recipients of HIV care at routine points-of-care in Uganda.

DTG-based ART was rolled out across Uganda in March 2018, this offered us an opportunity to explore perceptions of clinicians who have managed patients on DTG-based ART for at least 12 months [11, 24, 25].

### Analytical frame work

This study is broadly guided by an analytical framework proposed by Senkhon and colleagues [26] which is informed by a systematic review of the literature on the notion of acceptability of health care interventions. Based on this review, they concluded that acceptability is a multi-faceted concept and accordingly proposed seven dimensions of acceptability which include; Perceived effectiveness: This refers to the extent to which an intervention is perceived as likely to achieve its intended objective. Affective attitude: This denotes how an individual feels about the intervention, Burden: the amount of effort that is perceived as necessary to participate in an intervention Self efficacy: participants’ perceptions of the behavioural requirements of participating in an intervention and Opportunity cost: ‘The extent to which benefits, profits or values must be given up to engage in the intervention’ [26]. This framework guided data analysis by providing an overarching deductive thematic framework in which to categorize our inductively-generated sub-themes [27].

Although the notions acceptability and tolerability are often used interchangeably [26], with regard to tolerability, we adopted conceptual guidance from the definition advanced by the International Conference on Harmonization (ICH) “the degree to which overt adverse effects can be tolerated by the subject” [28]. This helped frame our findings on tolerability around perceived adverse drug reactions by participants.

### Study population

We sought three categories of participants to achieve our study objective of exploring acceptability and tolerability of DTG-based ART. The first category of participants were clinicians in ART clinics across Uganda. We sought to understand clinicians’ perspectives on the acceptability and tolerability of DTG based on their occupational experience of attending to patients taking DTG-based ART. We aimed for at least two participants from each of the 12 facilities for our in-depth interviews; the ART clinic manager and the most senior clinician. The second category of participants we sought were ART clinic managers to whom ADRs are reported and who routinely advise on the management of ADRs. Because ADRs are frequently reported to pharmacists especially at tertiary level of care in Uganda, the number of respondents at Regional Referral Hospitals included more than three participants on account of inclusion of pharmacists. The third category of informants we sought were national-level HIV programme managers (such as the Ministry of Health) who are privy to national-level policy agendas such on donor policies on DTG-based ART procurements since Uganda is heavily dependent on external donors for HIV treatment.

**Fig. 1:**
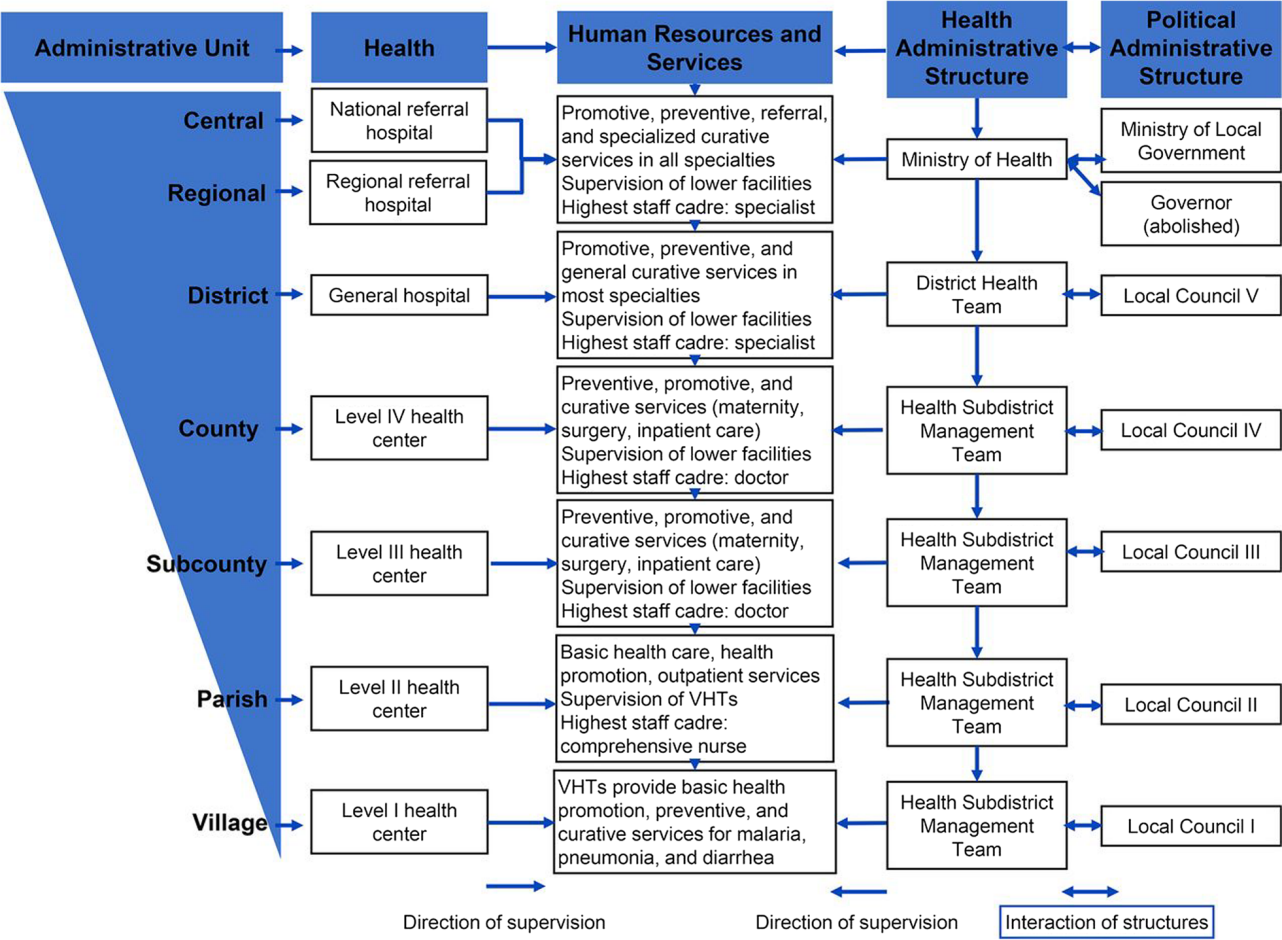
Level of service delivery

**Fig. 2:**
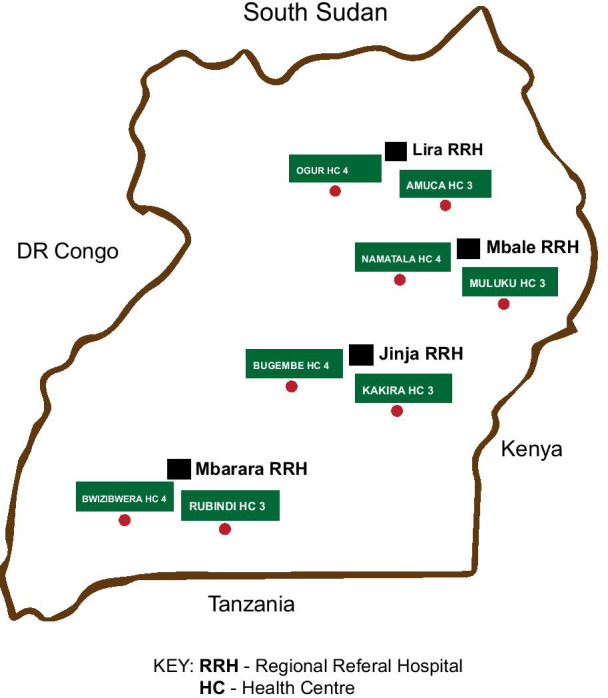
Study sites

With regard to clinicians in ART clinics, we sought a diverse sample representing multiple contextual settings [29] with regard to the different levels of service delivery in the Ugandan health-system (tertiary l/secondary/primary), as shown in Fig. 1, as well as across a range of cadres (medical doctor/clinical officer/nurse etc.) [29] and varied geographical sub-regions of Uganda (Northern, Eastern, Western and Central). To this end, a purposive sample of health facilities was selected. Figure 2 shows that we selected four Regional Referral Hospitals (RRHs) which were geographically representative of the four regions of Uganda. In each of the four regions of Uganda, we selected three health centres at the secondary level of care (Health Centre IVs) and primary care level (Health Centre IIIs).

We envisaged that this purposive sampling of health facilities would generate a diverse cadre of clinicians in ART clinics (e.g. medical doctors, clinical officers, nurses) who ordinarily attend to recipients of HIV care [29] and potentially encounter ADRs in the course of their routine practice or regular health services delivery. We selected at least three clinicians in the ART clinics of each of the 12 health facilities. The unit of participant selection was the ART clinic at each of the 12 participating facilities.

### Study setting

Uganda runs a largely vertical HIV care and treatment model since 2004 when President’s Emergency Plan for AIDS Relief (PEPFAR) commenced support [6]. Due to donor-funded vertical support, ART clinics run as stand-alone units solely dedicated to HIV service delivery within larger hospital complexes [6]. ART clinics run a parallel system for triage of patients with their own filing system. Typically, ART clinics have waiting areas with patient benches protected by an oversite tent. ART clinics usually have several rooms for clinicians and counsellors as well as a dedicated pharmacy unit. ART clinics have a dedicated workforce headed by a clinician or nurse depending on the level of care in the Ugandan health system (Fig. 1) with a handful of clinicians, counsellors and auxiliary staff including long-standing patients who serve as informal health workers [30]. ART clinics run on most days of the week and individual patients attend ART clinics every month or every quarter if they are clinically stable [30].

### Data collection

Between September 2020 and February 2021, we conducted a total of 22 In-depth interviews (IDIs) with clinicians, face-to-face at participating ART clinics, to elicit their perspectives on the acceptability and tolerability of DTG-based ART among recipients of HIV care and treatment. We particularly sought to interrogate their occupational experiences of HIV disease management using DTG-based ART at participating facilities. The in-depth interviews were conducted by the first author who has extensive experience in qualitative research with the assistance of two qualitative research assistants. These interviews, on average, lasted 45 to 60 min. All study participants signed a written informed consent prior to participating in the study.

#### Focus group discussions

In addition, we aimed to understand the perspectives of clinicians in ART clinics as a group rather than as individuals and deemed focus groups suitable for the purpose [31]. To this end, we conducted three focus group discussions (27 participants) in English with clinicians in the ART clinics of participating facilities who have managed patients taking DTG-based regimens for at least 12 months. To explore differences in manifestations of DTG-associated ADRs by level of care in the Ugandan health system, we conducted three focus groups with nine clinicians at each of the three levels of care in the Uganda health system (tertiary, secondary and primary care levels). We had nine participants in each of our three focus groups. On average each focus group lasted about 90 min. An approved focus group discussion (FGD) guide was used during the proceedings**.** The aim of the FGDs was to elicit the collective experiences of clinicians as a group [31] regarding the perceived acceptability and tolerability of DTG-based ART. We purposively selected clinicians at participating facilities who were in service at the onset of transitioning patients to DTG-based ART and could offer retrospective insights on medication safety. The focus groups were conducted by the first author who has extensive experience in qualitative health services research [32–34]. The first author was assisted by two research assistants who took notes during the proceedings and operated the recorder. The interviews were recorded only after securing oral consent from participants.

Furthermore, we conducted eight Key Informant Interviews (KIIs) with national-level HIV program managers seeking to understand national-level dynamics underpinning [35] the transitioning of patients to DTG-based ART such as donor programmatic targets. The key informants include four informants at the Ministry of Health headquarters charged with coordinating national HIV care and treatment services planning and four informants from the National Drug Authority responsible for national pharmacovigilance oversight.

### Data analysis

We utilized framework analysis as a data analysis approach which entails a systematic method of organizing and categorizing data based on key themes [27, 36]. Our data analysis approach was deductively-led by the underpinning analytical framework which proposes multiple dimensions of acceptability (e.g. perceived effectiveness, affective attitude, burden etc.) and the notion of tolerability which we operationalized as perceived adverse drug reactions of DTG-based ART from the perspective of participants. The research team took notes during field data collection and met every evening in peer debriefing sessions to consider analytical issues emerging from participant discourses.

The audio recordings of the interviews and focus groups were transcribed verbatim into text transcripts. The first author together with two co-authors read the transcripts multiple times to inductively identify themes as guided by the overarching deductive framework comprising of five themes (perceived effectiveness, affective attitude, burden, self-efficacy, opportunity costs). The emergent sub-themes relating to the acceptability and tolerability of DTG-based ART were then grouped under the deductive themes from the underpinning theoretical framework [27]. Disagreements with in the research team around categorization of data into thematic matrices were resolved through consensus (Additional file 1: Table S1).

## Results

### Participants by health worker cadre

Majority of the participants were clinical officers by cadre, followed by nurses and doctors. Participants included ‘expert patients’ or lay workers which is not uncommon at ART clinics across Uganda [29].

Table 1 shows the health worker cadres that participated in the study (n = 49).

**Table 1:** Cadre of health workers who participated in the study

Cadre of health worker	n
1. Medical doctors	6
2. Clinical Officers	15
3. Nursing cadre	10
4. Pharmacy cadre	4
5. Midwife	2
6. Data analysts	2
7. Laboratory technicians	2
8. Lay workers (‘expert patients’)	8
	49

In terms of gender, 51% of participating clinicians were male while 49% were female.

The findings emerging from this study are presented based the underpinning deductive thematic framework [26] shown in Table 2.

**Table 2:** Themes and sub-themes

Deductive Themes	Sub-themes
Acceptability	
Perceived effectiveness	Superior viral load suppression
Affective attitude	High acceptance of DTG-based ART
Burden	Reduced pill burden
	Less side effects
Self-efficacy	Improved portability of ARV packaging
Opportunity costs	Inconvenient timing of taking DTG-based ART
Tolerability (adverse events)	Hyperglycemia
	Insomnia
	Increased appetite and weight gain
	Reduced libido
	Perceived interactions between DTG and IPT

### Affective attitude

#### High acceptance of DTG-based ART

There was consensus among clinicians in ART clinics that DTG-based regimens were well tolerated by the majority of their patients. When clinicians were asked to estimate the percentage of patients who report DTG-associated ADRs, the most frequently mentioned percentage range was between 5 and 10%.

‘The majority of our patients are doing well on DTG and have no complaints. Actually, most of the patients prefer DTG. There are some who are unsatisfied with it (DTG). Some have mild side effects which they are tolerating. Many of these patients have been on drugs (ART) for some time. They have learnt to tolerate them (side effects)’ [ART clinic manger, Regional Referral Hospital, Western Uganda, female].

### Perceived effectiveness

Clinicians in ART clinics frequently mentioned that DTG had a superior viral suppression rate when compared with other regimens especially efavirenz-based ART. When probed on why they perceived this to be the case, clinicians pointed out several cases of patients whose viral load has not been adequately suppressed while on non-DTG regimens but were subsequently virally suppressed after being transitioned to DTG.

‘We had many patients in our clinic who were non-suppressors but became (viral) suppressors when we put them on DTG-based ART. In my experience of treating patients, DTG-based ART is better at viral load suppression’ [Clinician, Health Centre IV, Eastern Uganda, male].

### Burden

#### Less pill burden

Clinicians reported that patients were relieved at the reduced bill burden after being transitioned to DTG-based regimens. Although recipients of HIV care were previously required to swallow multiple tablets of antiretrovirals, with the introduction of DTG, they were only required to swallow a single tablet once-daily. One of the clinicians we talked to who was also living with HIV indicated that even the size of the DTG tablet was a lot smaller in size compared to earlier ones.

‘The pill burden has reduced. With DTG you only need to take one tablet a day. In years past, the volume of pills one had to take were intimidating. You literally swallowed pills of all colours. But with DTG it is just a single pill every day’ [Clinician, Health Centre IV, Eastern Uganda, female].

#### Less side effects

Clinicians in ART clinics reported that DTG actually had less side effects when compared with alternative regimens. It was frequently mentioned that patients experienced less side effects compared to efavirenz-based ART. The side effects that patients frequently mentioned as having diminished after switching from efavirenz-based regimens include dizziness, headaches and nightmares.

‘Actually most of the patients prefer DTG because of having less side effects. You know most of them here are farmers. So once they take it in the morning, they can even go to their gardens to till the land without feeling drowsy or the dizziness. Some like to take it in the morning because even if they don’t take food, at least they can go and dig in the morning and come back and eat so it has been good’. [Medical doctor, Regional Referral Hospital, Northern Uganda].

### Self-efficacy

#### Improved portability of ARV packaging

Clinicians reported that patients appreciated the improved portability of medication packaging. The once-daily, single tablet translated into a reduced load of medication to be carried home by patients. In addition, it was reported that the available medication package for DTG-based provided to patients in Uganda required less plastic bottles compared to alternative regimen packaging.

‘With this new (DTG-based ART) packaging of a 3-month supply in one tin they are happy. Patients carry only one tin in their pockets for (medication lasting) 3 months. Initially, if we were to dispense to patients for 6 months, it meant you needed six tins. Now they are just two tins, for 6 months’ [Clinician, Health Centre III, Western Uganda, female].

From the perspective of clinicians, the reduced weight and bulk of (DTG) medication packaging for patients helped reduce the risk of HIV-related stigma because medication packages were less conspicuous. Clinicians reported that patients appreciated the newer packaging as it reduced potential curiosity in the community of what load they carried in their bags. Furthermore, the reduced medication package bulk helped in instances where couples had not yet disclosed their HIV status to their long-term partners. Clinicians reported that patients who struggle with HIV sero-status disclosure found it easier to conceal medication packages from their partners which helped in their adherence to ART.

### Opportunity costs

#### Inconvenient timing

The majority of clinicians were in ART clinics based in predominantly rural settings and many of the patients they attend to derive their upkeep from agrarian livelihoods. Clinicians in ART clinics reported that patients complained that when they take DTG in the morning hours it weakens them physically yet they earn their living from tilling the land.

‘Patients tell us that from their experience, when they take the medicine in the morning they feel they don’t get enough (physical) energy to do their normal businesses. So they would prefer to take it after supper at night. They will say ‘I have a busy routine every morning. I would have to go to the kitchen first to prepare food every morning in order to swallow the medicine. I don’t have time’. [Clinician, Health Centre IV, Eastern Uganda, male].

Clinicians in ART clinics reported that due to early-morning occupational routines, many of the patients, contrary to instructions, take DTG-based ART at night for the first handful of days. It is only when patients experience insomnia they revert to taking DTG in the morning hours as advised.

### Tolerability

Several clinicians reported that a number of their patients experience suspected DTG-associated ADRs which was a significant concern for them as service providers.

#### Dolutegravir-induced hyperglycemia

Hyperglycemia was by far the most commonly cited DTG-associated ADR across our interviews with clinicians. Hyperglycemia was reported as a DTG-associated ADR in 10 (out of the 12) participating facilities. In this study, clinicians indicated that DTG-associated ADRs were highest at the tertiary level of care especially at the level of Regional Referral Hospitals. However, complaints of hyperglycemia were common in participant discourses. Hyperglycemia stood out prominently among DTG-associated ADRs.

‘The main issue with DTG is hyperglycemia. Some of my patients have been admitted in the ward with very high blood sugars. Today I have just removed a middle-aged woman from DTG and taken her back to efavirenz-based ART. They had a blood sugar reading of 20 mmol/dl’. [ART clinic manager, Health Centre IV, Eastern Uganda, male].

The majority of clinicians in ART clinics, including at the tertiary-level of care, reported that during the initial roll out of DTG which commenced in 2018, they did not conduct baseline blood glucose tests. This was attributed to the initial low awareness, among HWs, around the association between DTG and hyperglycemia. The shortage of glucometers and accompanying test kits in case study facilities was frequently cited by clinicians as a fundamental barrier to blood glucose monitoring for patients taking DTG-based ART.

‘There was no baseline or screening of blood sugars before transitioning patients. There were resource constraints. You need glucometers, test kits and all those kind of things and the IP (PEPFAR implementing partner) did not have a budget for that. So we are transitioning patients to DTG without baseline screening for diabetes’ [ART clinic manager, Regional Referral Hospital, Northern Uganda, male].

Due to commodity shortages, clinicians in the ART clinic indicated they frequently advise patients to make private out-of-pocket expenditure on blood glucose tests to rule out new-onset hyperglycemia. However, patients were reportedly often unable to raise the 5000 shillings (US $1.23) charged for tests by private providers.

Clinicians in ART clinics reported that health facilities were given programmatic targets for transitioning the majority of their patients to DTG-based ART by PEPFAR implementing organizations in their geographic sub-regions. In the case of the four participating Regional Referral Hospitals (3 out 4) had transitioned over 80% of their HIV client loads to DTG-based regimens. Lower-level facilities (particularly Health Centre IIIs) had a much lower rate of transition to DTG regimens (on average 30%). It emerged from participant discourses that although the programmatic targets set for them for transitioning patients to the more efficacious DTG-based regimens were understandable, there was insufficient attention paid to patient safety in roll-out targets.

The gap in blood glucose monitoring for patients on DTG was suspected by clinicians to have resulted in the deaths of three patients at two primary care facilities. Clinicians from these facilities indicated that in two of the fatalities, they discovered belatedly that the patients had undiagnosed underlying diabetic conditions after they had already been transitioned to DTG. The absence of a policy on compulsory blood glucose screening during the initial phase of DTG roll-out in 2018 and the shortage of associated commodities was suspected to have contributed to the escalation in the diabetic conditions of patients especially those that had been undiagnosed.

#### Insomnia

Insomnia was one of the most commonly reported suspected ADRs associated with DTG-based regimens by participants. Clinicians in ART clinics reported that patients complained of an inability to sleep at night after taking DTG. These reports were fairly consistent across our participants.

‘Lack of sleep at night is a common complaint among patients. Some people are affected and some are not affected. [Clinician, Regional Referral hospital, Northern Uganda, male].

It emerged from our interviews with clinicians in the ART clinic that complaints of insomnia were partly attributable to patients’ non-adherence to medication instructions. Although attending HWs advised patients to take DTG-based medication during morning hours, many of the patients were actually taking it at night as was the previous practice with efavirenz-based ART. This emerged as a potential contributory factor in the multiple cases of insomnia across case-study facilities.

‘Sometimes patients do not take our instructions seriously. You tell them to take this medicine in the morning hours and instead they take it at night. Later on they get those problems of insomnia and the others that is when they come and tell you ‘musawo’ (doctor) I have not been sleeping at night. Then you ask them when have you been taking this medicine? That’s when you learn they have been taking it at night’ [Pharmacist, Health Centre HC IV, Western Uganda, male].

Hence, patient behavior and practices in taking DTG-based regimens appeared to be of interest. For instance, it emerged that some patients preferred to take DTG-based regimen at night instead of the mornings due to HIV-related stigma at their workplaces.

‘HIV-related Stigma is it part of the problem. They fear to take ARVs (antiretrovirals) during the day when people are seeing them say in the offices or markets where they work’ [Clinician, Health Centre III, Eastern Uganda, female].

#### Increased appetite and weight gain

Increased appetite after being initiated on DTG was cited by multiple clinicians as a common complaint among patients. The medication was said to induce abnormal appetites in some patients during the course of DTG-based therapy.

‘We have had clients who complain about overeating. They have come and told me when they take the drug (DTG) the demand for food becomes abnormal. They tell me, “With this drug (DTG) you need to eat and eat. You eat several times”. In fact, for some patients when you want to transition them to DTG they tell you, “my friends tell me you need to have a lot of food, how am I going to manage?”. [Clinician, Health Centre IV, Eastern Uganda, female].

Weight gain among patients was another frequently cited side effect by clinicians in ART clinics. Although clinicians indicated that patients voluntarily report unexpected weight gain, they reported observing weight gain based on trends in weight readings in patients who had been taking DTG after multiple weeks.

#### Reduced libido in adult males

Clinicians reported that several adult males reportedly complain of reduced libido within a few weeks of being transitioned to DTG-based regimens. Several of the clinicians in ART clinics reported that they are approached by a number of adult males who complain of a low sex drive and a noticeable decline in interest in sex after transitioning to DTG. Clinicians recounted tales of male patients’ complaints from their regular female sexual partners of sexual dysfunction. Requests to revert to efavirenz-based regimens by male patients experiencing a low sex drive were cited across multiple case-study facilities.

‘The main problem that I have seen is loss of libido for men they say ‘omulilo gugenze’ (the fire has gone). Since I was put on DTG, I don’t feel like having sex. This new drug is affecting me’. It is true there is loss of libido. And you wonder whether to take the patient back to the former regimen and versus the recommended one but loss of libido has been there’ [ART clinic manager, Regional Referral Hospital, Eastern Uganda, female].

Participants reported that the prevalence of reduced libido post-transition to DTG may be higher than is actually reported to clinicians because several males do not open up due to fear of embarrassment owing to cultural notions of masculinity [37].

With regard to male patients who actually report reduced sex drive, clinicians reported that they frequently switch them back to their former regimens (more commonly efavirenz-based ART). Sex drive was said to improve in many cases after switching from DTG-based regimens.

“I have so far helped five males who developed low sex drive on transition to TLD, we changed them back to TLE (Tenofovir, Lamiduvine, Efavirenz) and now they are doing well” [ART clinic manager, Health Centre IV, Eastern Uganda, male].

#### Suspected drug interactions between DTG and Isoniazid Preventive Therapy

Clinicians reported that there were possible drug interactions between DTG-based ART and isoniazid preventive therapy (IPT) in the suspected ADRs reported by patients. It emerged that DTG was rolled out countrywide in 2018 and a year later, isoniazid preventive therapy (IPT)- was also rolled out nationally. Clinicians were emphatic in relaying the notion that initiating patients on these two medicines concurrently could result in ADRs. At a participating Regional Referral Hospital, clinicians reported 12 deaths of patients initiated on both DTG-based ART and IPT.

‘We have had over twelve deaths when I go back to a year ago (July 2019). These were patients who were on DTG-based ART and IPT. Some were emergencies referred to us from private providers and some coming from lower-level facilities. We would review them and find that they were on both of these drugs’ [ART clinic manager, Regional Referral Hospital, Western Uganda, female].

Clinicians in ART clinics however expressed extreme difficulty in disentangling ADRs associated with DTG-based ART from those attributable to IPT. Clinicians reported that whenever patients complained of suspected ADRs when they were newly initiated on both DTG-based ART and IPT, they were hard pressed in making causal links due to multiple limitations including a paucity of robust data on patients.

‘For the first 2 weeks they will come with complaints such as headache and many other issues. You find they are complaining of weakness and you reach somewhere you fail to understand which drug to stop or maintain. You ask yourself. Should I stop IPT or should I stop DTG-based ART? And it becomes a paradox’. [Clinician, Health Centre IV, Western Uganda, female].

It emerged from our interviews that during the initial roll-out of DTG-based ART in 2018 and IPT in 2019, concurrent introduction of both medications was permitted. However, subsequent guidance in the updated national ART treatment guidelines of 2020 advised against concurrent introduction of the two medications.

It emerged in this study that switching back to former regimens was a common strategy by clinicians whenever patients complained of suspected DTG-associated ADRs.

‘There was a 55-year old female. I transitioned her from efavirenz-based ART to DTG-based ART. On the third day she called me with all sorts of complaints; dizziness, headaches, general body weakness, a sore throat, she could not pass a fluid. I counselled her, that it might be due to another cause let her take it for a week but in the second week she returned it and she said ‘No. it is too much’ I discussed it with my pharmacy clinician. We put her back to efavirenz-based ART and she did well so after a week we were like we needed to record it as an adverse drug reaction’. [Clinician, Health Centre IV, Western Uganda, male].

Clinicians in ART clinics reported that in many instances ADRs cease once patients are switched back to efavirenz-based ART. However, clinicians complained of shortages of efavirenz-based ART due to a donor emphasis on stocking DTG-based regimens.

‘The challenge we have is that there is no efavirenz-based ART at our facility. When patients who react badly to DTG-based ART emerge, I start running here and there to get the drug. We begin running to nearby facilities to see if they have any. They are not stocking efavirenz-based ART anymore so what will we do when we need efavirenz-based ART?’ [Clinician, Health Centre IV, Eastern Uganda, female].

Across our interviews with clinicians, we observed that the higher the percentage of patients they transitioned to DTG-based ART, the more the cases of suspected ADRs they reported. For example, almost all four of the Regional Referral Hospitals (RRH)we visited had transitioned at least 80% of their patient populations to DTG-based regimens. RRHs also reported the highest cumulative number of suspected DTG-associated ADRs. Although this could be due to having significantly higher HIV client loads, the tertiary-level of care appeared to have noticeably higher cases of suspected ADRs associated with taking DTG-based ART.

## Discussion

We set out to explore the perceptions of clinicians in ART clinics in Uganda regarding the acceptability and tolerability of DTG-based ART since initial roll-out in March 2018. While clinicians in ART clinics acknowledged that DTG-based ART was well tolerated by the majority of their patients, they reported that a number of them suffer ADRs after being transitioned to the regimen. Hyperglycemia was the most commonly cited suspected ADR associated with DTG-based regimens. Insomnia, reduced libido and weight gain were among the other frequently cited suspected ADRs. Furthermore, clinicians in ART clinics perceived some ADRs as resulting from the concurrent use of DTG-based ART and isoniazid preventive therapy (IPT).

Dolutegravir-induced hyperglycemia was reported by clinicians from all (but two) of the 12 participating facilities. Clinicians from the two facilities that didn’t report hyperglycemia were lower-level facilities which had transitioned less than 30% of their HIV client loads to DTG-based regimes (compared to Regional Referral Hospitals which had transitioned over 80% of their patients to DTG). Further still, clinicians in ART clinics reported that at the onset of transitioning patients to DTG commencing in 2018, they did not carry out baseline blood glucose tests due to their initial lack of awareness around the association between DTG and hyperglycemia. Even when this association became apparent, the shortage of commodities for testing blood glucose such as glucometers and test kits were indicated as fundamental constraints at participating facilities. Uganda is heavily dependent on external donors for HIV treatment with PEPFAR being the primary source of international assistance [32–34]. Clinicians in ART clinics reported that regionally-based PEPFAR implementing organizations were not budgeting for blood glucose monitoring in their HIV response support programs. Furthermore, providers indicated that patients could not afford out-of-pocket expenditure on blood glucose tests at private outlets even when they were strongly recommended by attending clinicians. Whilst Uganda’s national ART guidelines of 2020 were updated to include blood glucose monitoring for patients on DTG-based regimens, supply chain barriers continue to hamper observance of these guidelines [38]. Although previous studies have reported hyperglycemia as a DTG-associated ADR [39–41], including a 2020 study in Uganda [25], our findings suggest that hyperglycemia could be more common than previously indicated. Further research utilizing more rigorous approaches and larger study samples is recommended. From a policy and programming lens, our study findings point to the need for increasing funding for blood glucose monitoring among HIV patients especially those on DTG-based regimens. In light of our findings, we add to the mounting calls for the integrated health services agenda especially around integrating HIV services with the management of non-communicable diseases (NCDs) such as with regard to the management of diabetes mellitus and hypertension [42, 43].

Insomnia was the second most cited suspected DTG-associated ADR in our sample of facilities. Although the nature of our research design did not allow us to make causal claims linking insomnia to DTG-based ART, our study suggests that the behavior of patients could be interest. More specifically, we found that it was common for patients to take DTG-based medication during night time contrary to explicit instructions from clinicians to take the tablet in the morning. A study by Nabitaka and colleagues [44] found that trouble sleeping was the third most prevalent self-reported side effect by patients who had been on DTG for at least 6 months. Previous studies have reported insomnia as an ADR associated with DTG [45–47], however our study shades more light on this phenomenon by suggesting that patient adherence to clinician instructions on when to take DTG-based ART is an attribute of interest. This calls for programming interventions aimed at improving adherence to medication instructions with regard to DTG-based regimens. Furthermore, our findings point to the need for increased sensitizations of patients prior to enrolling them on newer HIV medications [44, 48]. For instance, patients were used to a routine of taking efavirenz-based regimens at night. However, DTG-based regimens demanded a radical shift to taking ART medication in the morning. Importantly, the perspectives of clinicians suggest a link between patients deliberately taking ART medication at night contrary to the instructions of clinicians with HIV-related stigma or the fear by patients of taking this medication in the morning at the workplace in the presence of work colleagues [49, 50].

An important finding of this study is that deficiencies in the laboratory infrastructure and commodity stock availability (such as for blood glucose test kits) at frontline points-of care are a barrier to determining the prevalence of suspected DTG-associated ADRs which could be higher than can be determined at the facility-level due to health-system constraints [51, 52]. For instance, clinicians in ART clinics decried the lack of diagnostic tools and technologies for conducting regular liver function tests to identify suspected ADRs. This calls for increased investments in laboratory systems strengthening in Uganda to support early detection of suspected ADRs and prompt actions to secure patient safety for the over 1.2 million Ugandans enrolled on ART [32].

In this study, clinicians in ART clinics especially at the tertiary level of care indicated that they were under pressure to meet donor targets of transitioning the majority of their HIV client loads to DTG-based regimens. Although donors’ emphasis on transitioning to superior DTG-based regimens (such as in terms of viral load suppression) are understandable, it emerged that, from the perspective of providers, there has been insufficient attention paid to medication safety in the ambitious programmatic goals set for DTG transitions. In addition, HWs reported that they experienced shortage of efavirenz-based ART due to an on-going emphasis on transitioning to DTG-based regimens in Uganda. Clinicians in the ART clinics expressed concern that patients who experience DTG-associated ADRs and need to be switched to alternative regimens may not have these options available to them, which could severely affect their HIV treatment. Parkhurst and colleagues have observed the occasional tension between global health actor interests and donor-recipient national interests [53].

## Recommendations

Clinicians in the ART clinics suspected ADRs resulting from drug interactions between DTG and IPT. A hospital-based study by Griensven and colleagues [54] in Cambodia found that ‘IPT discontinuation due to drug toxicity was common in patients subsequently initiating ART’ highlighting the need for caution in initiating patients on both medications concurrently. The updated national ART treatment guidelines in Uganda of 2020 now advise against initiating patients on DTG and IPT concurrently. Wide dissemination and sensitizations of clinicians in ART clinics on these updated national HIV treatment guidelines is critical in ensuring patient safety from medication harm. It is common to find that lay health workers are engaged in clinical HIV disease management at ART clinics across Uganda [30]. Lay workers are a very diverse, informal group of health workers typically with a minimum of secondary school education who are co-opted on the staff of ART clinics in order to fill critical staffing shortages. This cadre of the workforce should be especially targeted for sensitizations and regular updates on the management of patients on DTG-based ART.

Additionally, the involvement of ‘expert patients’ in ART provision at participating facilities underlines the importance of regular trainings in pharmacovigilance and in recognizing ADRs for all personnel in ART clinics in Uganda [55]. A study in Uganda reports that task shifting to non-clinician cadres is as high as 93% [29, 30]. While health workforce shortages are undoubted, there is need for sensitizations on pharmacovigilance reporting for all who offer ART services in HIV clinics in Uganda which is listed by the WHO as one of the countries with a human resources for health crisis [29, 30, 55, 56].

## Limitations

This study had multiple limitations which we wish to acknowledge. Our study was conducted with clinicians in 12 purposively selected health facilities across Uganda. Qualitative research designs by their very nature have inherent limitations in statistical generalizability of findings. However, our primary objective was to enable an in-depth examination of the safety profile of DTG-based ART at the frontline level of service delivery and from the perspective of clinicians in ART clinics, given their operational contexts. In addition, it would perhaps have been more ideal to hear directly from the patients themselves or to learn from both clinicians and patients taking DTG-based regimens. In-depth interviews and focus groups with patients are planned in a subsequent component of this study.

## Conclusion

While DTG-based regimens were perceived by clinicians in ART clinics to be well tolerated by the majority of their patients, they also reported that a number of their patients experience suspected ADRs key among which were hyperglycemia, insomnia and reduced libido. Based on the perspectives of clinicians, we recommend that future studies examine the prevalence of dolutegravir-induced hyperglycemia in patients in Uganda.

## Supplementary Information

**Additional file 1: Table S1.** The consolidated criteria for reporting qualitative studies (COREQ): A 32-item(s) checklist.

## Data Availability

The datasets generated during and/or analyzed during the current study are not publicly available due to ethical reasons but are available from the corresponding author on reasonable request.

## Abbreviations

AIDS: Acquired Immune Deficiency Syndrome
ADRs: Adverse Drug Reactions
ART: Anti-retroviral therapy
ARVs: Anti-retrovirals
DTG: Dolutegravir
MOH: Ministry of Health
PEPFAR: The Presidents' Emergency Plan for AIDS Relief
RA: Research Assistant
SSA: Sub-Saharan Africa
TLD: Tenofovir, Lamivudine, and Dolutegravir
TLE: Tenofovir, Lamivudine, and Efavirenz
WHO: World Health Organization
